# Synthesis and Biological Testing of Novel Glucosylated Epigallocatechin Gallate (EGCG) Derivatives

**DOI:** 10.3390/molecules21050620

**Published:** 2016-05-11

**Authors:** Xin Zhang, Jing Wang, Jiang-Miao Hu, Ye-Wei Huang, Xiao-Yun Wu, Cheng-Ting Zi, Xuan-Jun Wang, Jun Sheng

**Affiliations:** 1 Key Laboratory of Pu-er Tea Science, Ministry of Education, Yunnan Agricultural University, Kunming 650201, China; jeachris@sina.com (X.Z.); wangjingzb4321@163.com (J.W.); lichuangyewei100@163.com (Y.-W.H.); wuxiaoyun79@163.com (X.-Y.W.); 2 Tea Research Center of Yunnan, Kunming 650201, China; 3 Pu-er Tea Academy, Yunnan Agricultural University, Kunming 650201, China; 4 State Key Laboratory of Phytochemistry and Plant Resources in West China, Kunming Institute of Botany, Chinese Academy of Sciences, Kunming 650201, China; hujiangmiao@mail.kib.ac.cn; 5 State Key Laboratory for Conservation and Utilization of Bio-Resources in Yunnan, Kunming 650201, China

**Keywords:** epigallocatechin gallate (EGCG), glycosides, anticancer activity, DPPH, synthesis

## Abstract

Epigallocatechin gallate (EGCG) is the most abundant component of green tea catechins and has strong physiological activities. In this study, two novel EGCG glycosides (EGCG-G1 and EGCG-G2) were chemoselectively synthesized by a chemical modification strategy. Each of these EGCG glycosides underwent structure identification, and the structures were assigned as follows: epigallocatechin gallate-4′′-*O*-β-d-glucopyranoside (EGCG-G1, **2**) and epigallocatechin gallate-4′,4′′-*O*-β-d-gluco-pyranoside (EGCG-G2, **3**). The EGCG glycosides were evaluated for their anticancer activity *in vitro* against two human breast cell lines (MCF-7 and MDA-MB-231) using MTT assays. The inhibition rate of EGCG glycosides (EGCG-G1 and EGCG-G2) is not obvious. The EGCG glycosides are more stable than EGCG in aqueous solutions, but exhibited decreasing antioxidant activity in the DPPH radical-scavenging assay (EGCG > EGCG-G2 > EGCG-G1). Additionally, the EGCG glycosides exhibited increased water solubility: EGCG-G2 and EGCG-G1 were 15 and 31 times as soluble EGCG, respectively. The EGCG glycosides appear to be useful, and further studies regarding their biological activity are in progress.

## 1. Introduction

**Figure 1:**
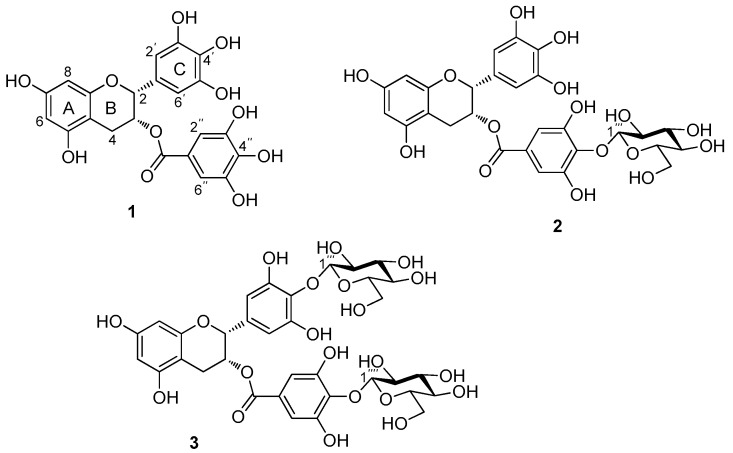
Structures of EGCG (**1**), EGCG-G1 (**2**), and EGCG-G2 (**3**).

Green tea has been consumed by human beings for thousands of years and is an important source of tea catechins. Tea catechins, including catechin (C), epicatechin (EC), gallocatechin (GC), epigallocatechin (EGC), epicatechin-3-gallate (ECG), gallocatechin-3-gallate (GCG), and epigallocatechin gallate (EGCG), have many reported biological activities, such as antioxidative, anticarcinogenic and antitumor properties [1,2,3]. Specifically, epigallocatechin gallate (EGCG, **1**, Figure 1) is the most abundant catechin (accounting for approximately 50% of total catechins) [4] and has been reported to have stronger physiological activities than others [5,6,7,8]. EGCG has been studied previously for its effects in inducing breast cancer apoptosis and cell-cycle arrest [9,10]. Another study also suggested that EGCG could be used as an agent to block smoking (Nic)- or hormone (E2)-induced breast cancer cell proliferation by inhibiting the α9-nAChR signaling pathway [11] and could block Wnt signaling by inducing the HBP1 transcriptional repressor to inhibit aspects of invasive breast cancer [12]. These mechanisms reveal that EGCG may have significant chemopreventive applications in human breast cancer. Accordingly, substantial attention has been paid to tea catechins for their potential applications.

However, the use of EGCG is often hindered by problems such as its poor water solubility [13], rapid metabolism [14], and ready degradation in aqueous solutions [13]. To obtain more potent analogues and overcome this problem, a great deal of research has been performed. Examples include permethyl EGCG [15], peracetyl EGCG [16,17], perbenzylated EGCG [18], EGCG-octa-*O*-TBDMS [19], EGCG-3SH [20], EGCG monoester derivatives [21], and EGCG glycosides [13,22,23,24], which are more active than EGCG or have been found to inhibit EGCG oxidation. Potential anticancer agents such as the reported EGCG derivatives are often associated with undesirable side effects, including decreases in the water solubility and stability of EGCG. Thus, the structural modification of EGCG to develop new compounds with increased solubility and stability is highly desirable. As illustrated in the literature [13,22,23,24,25], the glycosylated derivatives of catechins exhibited similar antioxidant properties, increased solubility in water and stronger tyrosinase inhibitory effects. Thus, the transglycosylated derivatives of EGCG are important for their utility as food additives and cosmetics. 

Recently, preparation of glycoconjugates of small molecule drugs has become an attractive strategy in order to overcome problems of water solubility and stability in aqueous solution and reduce side effects [26,27]. Both epigallocatechin gallate-5-*O*-α-d-glucopyranoside [28] and epigallocatechin gallate-4′,4′′-*O*-α-d-glucopyranoside [28,29], two EGCG glycosides have been synthesized by enzymatic reactions, are derivatives of EGCG α-d-glucopyranoside that exhibit similar antioxidant effects and are more water soluble than EGCG. The corresponding β-d-glucopyranosides of EGCG are not reported in the previous literature. The chemical modification by glycosylation is still not known for EGCG, and the chemical modification of compounds can be controlled with respect to biosynthesis and should further broaden the potential industrial applications. Therefore, the chemical modification of EGCG may be an effective strategy for synthesizing useful epigallocatechin gallate glycosides.

In this study, we report the synthesis of EGCG glycosides by a chemical strategy, in which a d-glucopyranosyl residue is attached to the 4′′-hydroxyl group of the D-ring (EGCG-G1, **2**) or both the 4′- and 4′′-hydroxyl groups of EGCG (EGCG-G2, **3**, Figure 1). The glycosylated epigallocatechin gallate derivatives were screened for biological activities, including antitumor activity and antioxidant activity.

## 2. Results and Discussion

### 2.1. Chemical Synthesis and Structural Determination of EGCG Glycosides

**Figure 2:**
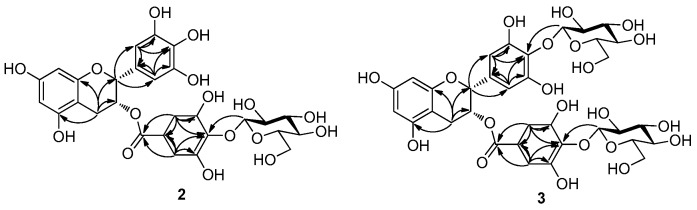
HMBC correlations of EGCG-G1 (**2**) and EGCG-G2 (**3**).

The molecular ion of EGCG-G1 was observed at *m*/*z* 619 (M − H)^−^, indicating a molecular formula C_28_H_28_O_16_ confirmed by HRESIMS (*m*/*z* 619.1295 [M − H]^−^, calcd for 620.1377); mp. 182–185 °C; ${\left[\alpha \right]}_{D}^{23.8}$: −10.8 (*c* 0.12, DMSO). In its ^1^H-NMR spectrum (Table 1), the doublet signal at 4.87 ppm (d, *J* = 9.0 Hz) was assigned to the anomeric proton. In addition, C-1′′′ was observed at 106.0 ppm in the ^13^C-NMR spectra, showing that only one glucosyl residue is β-linked to EGCG. In the HBMC data (Figure 2), the chemical shift of C-4′′ was observed at 137.1 ppm, and coupling appeared to have occurred between C-4′′ and H-1′′′ (4.87 ppm, d, *J* = 9.0 Hz) of the glucosyl residue, which indicated that the glucosyl residue had been attached to C-4′′ of the EGCG, whereas C^2^′′-H, C^6^′′-H (6.82 ppm) occurred with C-2, C-1′′, C-3′′, C-4′′ and C-5′′ of the D ring; C2′-H, C6′-H (6.52 ppm) occurred with C-1′, C-3′′, C-4′′ and C-5′′ of the C ring; H-4 was observed at 2.89–2.69 ppm and 2.58–2.49 ppm, and its couplings occurred with C-2, C-3, C-5 and C-10 of EGCG. Based on these results, we determined that the structure of EGCG-G1 could be most appropriately referred to as epigallocatechin gallate-4′′-*O*-β-d-glucopyranoside (**2**, Figure 1).

The molecular ions of EGCG-G2 was observed at *m*/*z* 781 (M − H)^−^, having a molecular formula determined as C_34_H_38_O_21_ by HRESIMS (*m*/*z* 781.1814 [M − H]^−^, calcd for 782.1906); mp. 208–212 °C; ${\left[\alpha \right]}_{D}^{23.7}$: −67.1 (*c* 0.15, DMSO) In Table 1, the two doublet signals at 4.70 ppm (d, *J* = 9.5 Hz) and 4.46 ppm (d, *J* = 9.5 Hz) were assigned to the anomeric protons, showing that two glucosyl residues are β-linked to the EGCG.

Based on the HMBC data (Figure 2), coupling occurred between H-1′′′ (4.70 ppm, *J* = 9.5 Hz) of the glucosyl residue and C-4′ (135.6 ppm) of the C ring and also between H-1′′′′ (4.46 ppm, *J* = 9.5 Hz) of the other glucosyl residue and C-4′′ (137.1 ppm) of EGCG. These signals indicated that the two glucoses had been attached to the C-4′ and C-4′′ of EGCG. The chemical shift for the proton at C^2^′′-H, C^6^′′-H (6.80 ppm) exhibited coupling with C-2, C-C-1′′, C-3′′, C-4′′ and C-5′′ of EGCG; C^2^′-H, C^6^′-H (6.52 ppm) occurred with C-1′, C-3′′, C-4′′ and C-5′′ of the C ring; whereas H-4 was observed at 2.99–2.89 ppm and 2.70–2.66 ppm, and its couplings occurred with C-2, C-2, C-5 and C-10 of EGCG. These results indicate that two β-glucosidic linkages (β-1′′′→4′ and β-1′′′′→4′′) formed during the reaction, and EGCG-G2 could be most appropriately referred to as epigallocatechin gallate-4′,4′′-*O*-β-d-glucopyranoside (**3**, Figure 1).

### 2.2. Anticancer Activity

**Figure 3:**
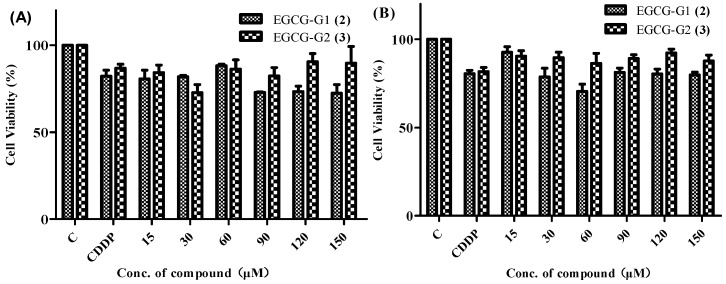
*In vitro* anticancer activity of EGCG glycosides: EGCG-G1 (**2**) and EGCG-G2 (**3**). MTT cell viability assays of human breast cancer cell lines MCF-7 (**A**) and MDA-MB-231 (**B**) treated with cisplatin (CDDP), EGCG-G1 (**2**) and EGCG-G2 (**3**). The cells were seeded in 96-well plates and, after overnight incubation, treated with various agent concentrations for 48 h. The viability values are represented as percentages with respect to the untreated cells (regarded as 100% viability). The bars demonstrate the means of triplicate experiments with SD.

The EGCG glycosides EGCG-G1 and EGCG-G2 were tested for their anticancer activity against two human breast cancer cell lines, MCF-7 and MDA-MB-231. Cisplatin (CDDP) was used as a reference compound. The screening procedure was based on the standard MTT method [30], Figure 3 illustrates the cell viability results, while treatment of EGCG (20 μM) for 48 and 72 h markedly increased the percentage of apoptotic cells (32%–51%) in MCF-7 cells compared to that of non-EGCG treated cells (8%–14%) [31], treatment of EGCG (20 μM) for 48 h and 72 h increased the percentage of apoptotic cell (71%–75%) in MDA-MB-231 [32]. The EGCG glycosides show weak activity against the two cancer cell lines tested compared with EGCG. As shown in Figure 3, the concentration of EGCG-G2 (25.56 μM) with the lowest of cell viability is 74% whereas EGCG-G1 (64.52 μM) is 69%. From these results, the inhibition rate of EGCG glycosides (EGCG-G1 and EGCG-G2) is not obvious.

### 2.3. Antioxidant Activity

**Figure 4:**
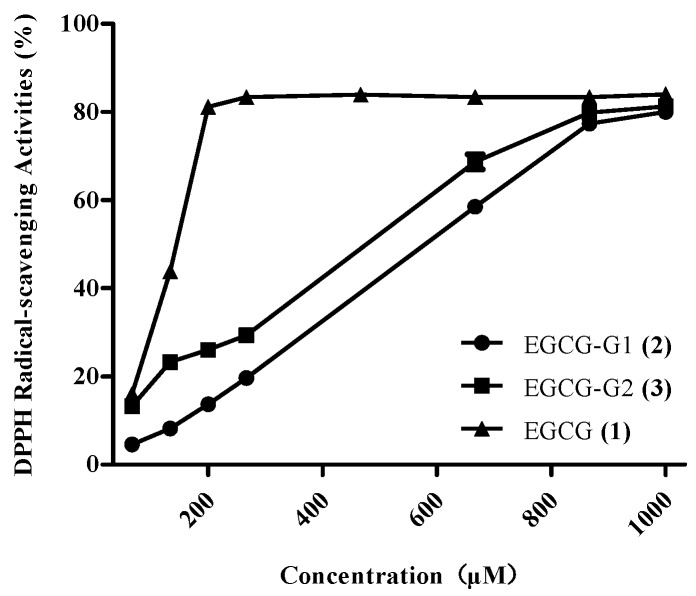
DPPH radical–scavenging activities of EGCG and EGCG glycosides: EGCG-G1 (**2**) (100 μL of 4.04, 8.07, 16.13, 24.20, 241.95, 483.90, 873.60 μM), EGCG-G2 (**3**) (100 μL of 3.20, 6.39, 12.78, 19.17, 191.70, 383.40, 511.20 μM) or EGCG (100 μL of 5.46, 10.92, 21.84, 32.76, 327.60, 655.20, 870.4 μM) was mixed with 0.13 μM 1,1-diphenyl-2-picrylhydrazyl (100 μL) in darkness at room temperature for 30 min, and the absorbance was monitored at 519 nm. Each value is the mean ± standard deviation (*n* = 3).

DPPH is a stable free radical that has been widely used to determine antioxidant capacity. Figure 4 shows the DPPH radical scavenging activities of EGCG and EGCG glycosides. Our first observation is that the compounds with glucose residues (EGCG-G1, EGCG-G2) mostly show lower activity than EGCG. Previously, Rise [33] and Moon [29] reported the antioxidant activities of EGCG glycosides. Moon and Lee [22] reported that the 4-hydroxy moiety in the C ring plays an important role in DPPH radical scavenging. We found that the DPPH radical scavenging activity ranking of EGCG and EGCG glycosides was EGCG > EGCG-G2 > EGCG-G1. According to Nanjo [34], the EGCG glycoside epigallocatechin gallate-4′-*O*-α-d-glucoside exhibited lower antioxidant activity than epigallocatechin gallate-4′,4′′-*O*-α-d-glucoside. These values showed a pattern similar to that of our results. Thus, we confirmed that the *O*-trihydroxyl group in the C ring and the galloyl moiety are the most important structural features for scavenging activity in the DPPH radicals.

### 2.4. Stability Investigation

**Figure 5:**
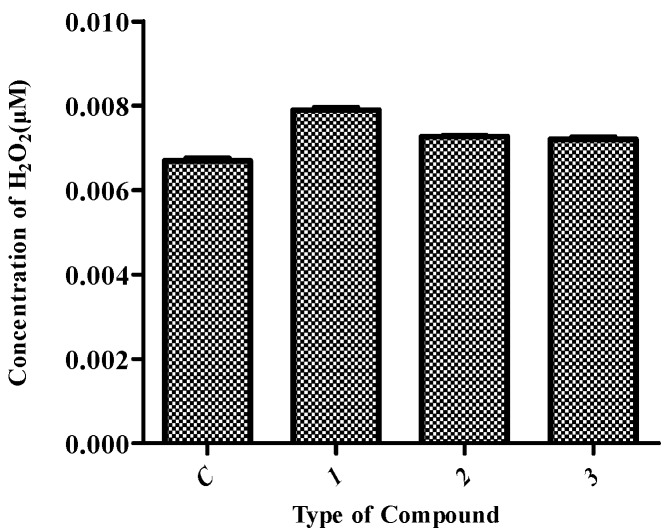
H_2_O_2_ concentrations of EGCG and EGCG glycosides: EGCG (**1**), EGCG-G1 (**2**) and EGCG-G2 (**3**), tested using a H_2_O_2_ Quantitative Assay Kit (Water-Compatible).

Hydrogen peroxide (H_2_O_2_) is a by-product of biological oxidation processes and has been used as a method for investigating the stability of compounds. Figure 5 shows the H_2_O_2_ concentrations of the three compounds EGCG, EGCG-G1 and EGCG-G2. EGCG is readily degraded in aqueous solutions, whereas the EGCG glycosides EGCG-G1 and EGCG-G2 are more stable. This result indicates that the glucose residue is significant for the EGCG derivatives, but the effect of the number of glucosyl residues attached to EGCG shows no significant difference in this study. For example, the H_2_O_2_ concentration of EGCG-G1 (0.007 μM) is nearly the same as for EGCG-G2.

### 2.5. Effects of Glycosylation on Water Solubility

We also determined the water solubilities of the EGCG and EGCG glycosides. The solubility of EGCG was 16.05 mM, whereas the solubilities of EGCG-G1 and EGCG-G2 were 240.93 mM and 504.73 mM, or 15 and 31 times higher than for EGCG. Table 2 shows the increase in the water solubility of the EGCG glycosides and that the number of glycol units improved the aqueous solubility of the compounds.

## 3. Experimental Section

### 3.1. General Information

d-Glucose was purchased from Sinopharm Chemical Reagent Co., Ltd. (Shanghai, China). EGCG was obtained from Chengdu Biopurify Phytochemicals, Ltd. (Chengdu, China). 3-(4,5-Dimethyl-thiazol-2-yl)-2,5-diphenyltetrazolium bromide (MTT) was obtained from Sigma Aldrich (St. Louis, MO, USA). Quantitative Assay Kit was purchased from Sangon Biotech Co., Ltd. (Shanghai, China). H_2_O_2_ Quantitative Assay Kit was purchased from Sangon Biotech Co., Ltd. The ZORBAX SB-C_18_ HPLC column was purchased from Agilent Technologies Co., Ltd. (Guangzhou, SC, China). KQ-300DE ultrasonic cleaner was purchased from Kun Shan Ultrasonic Instruments Co., Ltd. (Jiangsu, China). MCF-7 and MDA-MB-231 were purchased from the American Type Culture Collection (Rockville, MD, USA). All other chemicals used were of analytical grade. MS data were obtained in the ESI mode on an Qstar Pulsar instrument (API, Manchester, UK). HRMS data were obtained in the ESI mode on an LCMS-IT-TOF instrument (Shimadzu, Kyoto, Japan). NMR spectra were acquired on a Bruker AV-400 or DRX-500 instrument (Bruker BioSpin GmbH, Rheinstetten, Germany), using tetramethylsilane (TMS) as an internal standard. Column chromatography (CC) was performed on flash silica gel (200–300 mesh; Qingdao Makall Group Co., Ltd.; Qingdao, China). All reactions were monitored using thin-layer chromatography (TLC) on silica gel plates.

### 3.2. General Procedure for the Synthesis of EGCG-G1 and EGCG-G2

d-Glucose (18 g, 100 mmol) was suspended in acetic anhydride (100 mL), and sodium acetate (11 g, 130 mmol) was added with heating and stirring at 100 °C for 20 min. The mixture was cooled, poured into 500 mL of ice water and stirred for 1 h. The product was washed with aqueous saturated sodium bicarbonate solution and brine, dried, and evaporated to a syrup, from which the pentacetate crystallized from 95% alcohol. Next, the crystals were treated with a 33% (*w*/*w*) solution of hydrobromic acid in acetic acid (90 mL, 0.5 mmol). After 90 min, the solution was diluted with dichloromethane (150 mL) and washed with ice-cold water (3 × 150 mL), followed by aqueous saturated sodium bicarbonate solution (150 mL). The organic phase was dried with anhydrous magnesium sulfate, filtered and concentrated to afford crude 2,3,4,6-tetra-*O*-acetyl-α-d-glucopyranosyl bromide as a white solid (38 g, 94%) [35]. EGCG (1 g, 2.2 mmol) was dissolved in acetone (10 mL), and potassium carbonate (0.6 g, 4.3 mmol) was added with slow stirring over 30 min. Then, glucosyl bromide (0.9 g, 2.2 mmol) was added with heating and stirring at 55 °C for 12 h. The mixture was cooled and filtered, the filtrate was concentrated and dried in vacuo overnight. A potassium hydroxide solution (1.2 mmol dissolved in 3 mL of H_2_O) was added to an ice-cooled solution of crude product in CH_3_OH (3 mL). The mixture was stirred at 0–4 °C for 3 days and then neutralized with Dowex 50WX4-400 ion-exchange resin to pH 7. The solvent was evaporated, and the residue was purified by column chromatography (CHCl_3_/CH_3_OH/H_2_O, 64%:31%:0.5%) to afford the major products EGCG-G1 (110 mg, 11%) and EGCG-G2 (270 mg, 27%). The synthesized EGCG glycosides were characterized by ^1^H-NMR, ^13^C-NMR (results are summarized in Table 1), ^1^H-COSY, HSQC, HMBC, ESI-MS, and HRESI-MS (see Figures S1–S14 for more details).

### 3.3. Cell Culture and Cytotoxicity Assay

The following human breast cancer cell lines were used: MDA-MB-231 and MCF-7. All cells were cultured in RMPI-1640 or DMEM medium (Hyclone, Logan, UT, USA) supplemented with 10% fetal bovine serum (Hyclone) at 37 °C in a humidified atmosphere with 5% CO_2_. Cell viability was assessed by conducting colorimetric measurements of the amount of insoluble formazan formed in living cells based on the reduction of MTT. Briefly, cells were plated at a density of 20,000 cells/well in 96-well plates. After overnight incubation, the cells were treated with different concentrations of EGCG and EGCG glycosides, EGCG (**1**), EGCG-G1 (**2**) and EGCG-G2 (**3**), for 48 h. After incubation for specified times at 37 °C in a humidified incubator, 20 μL of MTT (5 mg/mL in PBS solution) was added to each well, and the cells were incubated for a further 4 h. After removal of the medium, 150 μL of DMSO was added to each well. The absorbance was recorded on a microplate reader at the wavelength of 490 nm. The effects of the agent(s) on growth inhibition were assessed as percent cell viability, where the untreated cells were taken as 100% viability [30]. 

### 3.4. DPPH Radical-Scavenging Assay

DPPH free radical scavenging capability was tested by the method described by Hung [36]. First, DPPH (10 mg) was mixed with ethanol (200 mL). After storage at room temperature for 30 min, the absorbance of the reaction mixture was recorded at 519 nm against a blank. The percentage of inhibition of DPPH radical-scavenging activity (*I*%) was calculated according to the following equation: *I*% = [(*A*_blank_ − *A*_sample_)/*A*_blank_] × 100, where *A*_sample_ is the absorbance of a sample solution, and *A*_blank_ is the absorbance of the blank solution (containing all reagents except the test sample). The IC_50_ value is the effective concentration that could scavenge 50% of the DPPH radicals.

### 3.5. Stability Assay

The H_2_O_2_ concentrations of EGCG and the EGCG glycosides, EGCG (**1**), EGCG-G1 (**2**) and EGCG-G2 (**3**), were detected using the H_2_O_2_ Quantitative Assay Kit (Water-Compatible) (Sangon Biotech Co., Ltd.). Briefly, 20 μL samples and 100 μL of caspase reagents were added to each well of a 96-well microtiter plate (Thermo Scientific, Waltham, MA, USA) and incubated for 20 min on a rotary shaker at room temperature. The absorbance was recorded on a microplate reader at the wavelength of 595 nm.

### 3.6. Water Solubility Analysis

The concentrations of the EGCG and EGCG glycosides were calculated as described by Li’s method [37]. All compounds were mixed in 200 μL of water in an Eppendorf tube at room temperature. A KQ-300DE ultrasonic cleaner was used to maximize solubility. After 1 h of sonication, each sample was diluted and then filtered through a 0.45 μm MFS membrane for HPLC analysis to determine the concentrations. Analytical HPLC was performed on an Agilent 1100 liquid chromatograph equipped with a ZORBAX SB-C_18_ (4.6 × 250 mm) column. The detection wavelength was 280 nm. The injection volume was 10 µL. The flow rate was 1 mL/min. Mixed solvents of methanol and water (30% for EGCG **1**, 23% for EGCG-G1 **2**, 45% for EGCG-G2 **3**) were used as the mobile phase.

### 3.7. Statistical Analysis

Data were analyzed by SPSS and expressed as the mean ± standard deviation (SD) for at least three replicates for each sample. A significant difference was evaluated at a level of *p* < 0.05.

## 4. Conclusions

In summary, we have used d-glucose and epigallocatechin gallate (EGCG) in a chemical modification strategy to synthesize two novel glycosylated EGCG derivatives EGCG-G1 and EGCG-G2 and characterized their structures. EGCG-G1 (**2**) contains one β-glucosidic linkage (β-1′′′→4′′) on the D ring of EGCG, and EGCG-G2 (**3**) contains two β-glucosidic linkages (β-1′′′→4′ and β-1′′′′→4′′) on the C and D rings, respectively. The EGCG glycosides have been assessed for their anticancer activities against two human breast cancer cell lines (MCF-7, MDA-MB-231). The inhibition rate of EGCG glycosides (EGCG-G1 and EGCG-G2) is not obvious. The EGCG glycosides exhibited decreasing antioxidant activity by DPPH radical-scavenging assay (EGCG > EGCG-G2 > EGCG-G1). Furthermore, EGCG glycosides show increased water solubility, and increasing the number of glycol units improved the aqueous solubility. Thus, our results suggest that some of these EGCG glycosides will be more useful as food additives and cosmetics. In addition, EGCG derivatives have the potential for further development as anticancer agents by structure modification.

## Figures and Tables

**Table 1:** ^1^H-NMR and ^13^C-NMR data of EGCG-G1 and EGCG-G2 in DMSO-*d*_6_^a^.

C Position	EGCG-G1 (δ_1_)		EGCG-G2 (δ_2_)	
	δ_C_	δ_H_	δ_C_	δ_H_
EGCG				
2	77.3	5.03 (s)	77.2	5.03 (s)
3	69.3	5.37 (br s)	69.4	5.38 (br s)
4	25.6	2.89–2.69 (m)	25.6	2.99–2.89 (m)
		2.58–2.49 (m)		2.70–2.66 (m)
5	156.7		156.7	
6	95.7	5.94 (d, *J* = 2.4 Hz)	95.7	5.94 (d, *J* = 2.4 Hz)
7	155.3		155.3	
8	94.2	5.84 (d, *J* = 2.4 Hz)	94.3	5.84 (d, *J* = 2.4 Hz)
9	156.5		156.5	
10	92.0		92.1	
1′	125.6		125.6	
2′/6′	105.6	6.52 (s)	105.8	6.51 (s)
3′/5′	150.3		150.3	
4′	135.6		135.6	
1′′	132.6		132.6	
2′′/6′′	108.6	6.82 (s)	108.6	6.80 (s)
3′′/5′′	149.8		149.8	
4′′	137.1		137.1	
COO-	164.7		164.7	
Glucose (-G1)				
1′′′	106.0	4.87 (d, *J* = 9.0 Hz)	106.0	4.70 (d, *J* = 9.5 Hz)
2′′′	75.1	3.33–3.15 (m)	75.8	3.30–3.22 (m)
3′′′	76.4	5.25–5.24 (m)	76.7	5.14–5.13 (m)
4′′′	73.6	3.33–3.15 (m)	73.6	3.30–3.22 (m)
5′′′	77.2	4.59–4.57 (m)	77.1	4.56–4.55 (m)
6′′′	60.4	3.60–3.58 (m)	60.4	3.61–3.57 (m)
Glucose (-G2)				
1′′′′			104.6	4.46 (d, *J* = 9.5 Hz)
2′′′′			75.7	3.30–3.22 (m)
3′′′′			76.1	5.24–5.23 (m)
4′′′′			73.6	3.30–3.22 (m)
5′′′′			77.1	4.61–4.60 (m)
6′′′′			61.2	3.61–3.57 (m)

^a^ ^1^H-NMR (500 MHz), ^13^C-NMR (125 MHz).

**Table 2:** Solubilities of EGCG and EGCG glycosides.

Compound	Solubility in Water ^a^ (mM)	Relative Solubility
EGCG	16.05 ± 1.23	1
EGCG-G1	240.93 ± 1.91	15
EGCG-G2	504.73 ± 0.57	31

a. Mean ± standard deviation (*n* = 3).

## Supplementary Materials

Supplementary materials can be accessed at: http://www.mdpi.com/1420-3049/21/5/620/s1.
