# Supplementary material for: Synthesis and Biological Testing of Novel Glucosylated Epigallocatechin Gallate (EGCG) Derivatives
Source: Molecules. 2016 May 11;21(5):620. doi: 10.3390/molecules21050620 (PMC6274015; doi:10.3390/molecules21050620)
Supplement: Supplementary file 1 [file molecules-21-00620-s001.pdf]

Supporting Materials: Synthesis and Biological Testing of Novel Glucosylated Epigallocatechin Gallate (EGCG) Derivatives

Xin Zhang, Jing Wang, Jiang-Miao Hu, Ye-Wei Huang, Xiao-Yun Wu, Cheng-Ting Zi, Xuan-Jun Wang, Jun Sheng

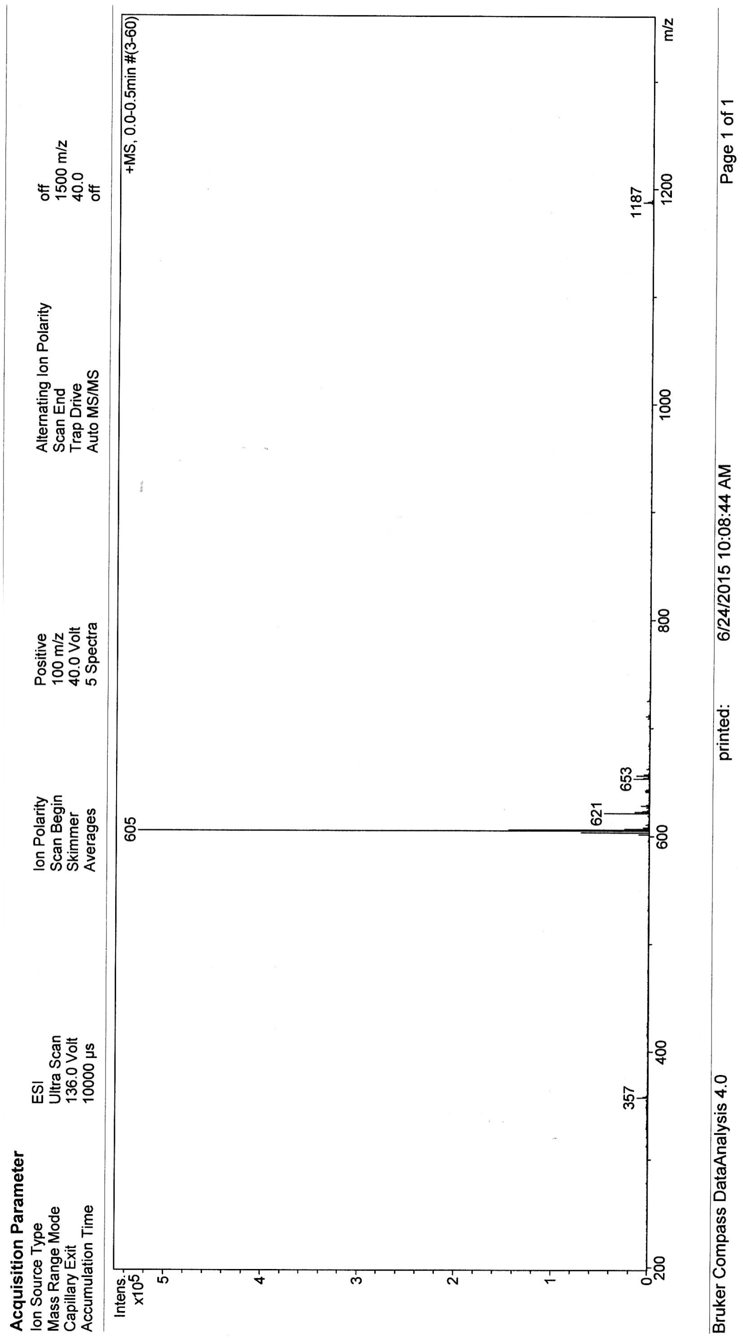

Figure S1. ESI MS of EGCG-G1 (**2**).

User Spectra

Fragmentor Voltage	Collision Energy	Ionization Mode
135	0	ESI

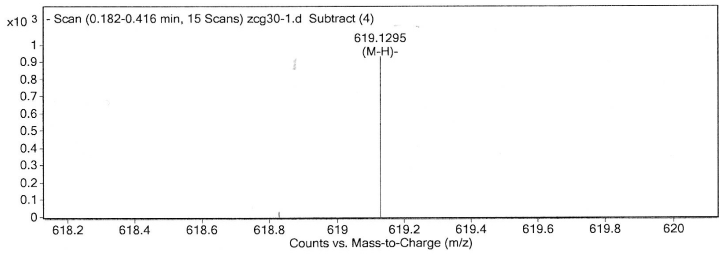

Peak List

m/z	z	Abund
487.07	1	1200.73
661.1404	1	1999.82
697.1187	1	946.73
717.1296	1	4251.5
718.1335	1	1604.54
759.1401	1	4544.87
760.1435	1	1826.15
775.1333	1	2354.84

Formula Calculator Element Limits

Element	Min	Max
C	3	60
H	0	120
O	0	20

Formula Calculator Results

Formula	CalculatedMass	CalculatedMz	Mz	Diff. (mDa)	Diff. (ppm)	DBE
C28 H28 O16	620.1377	619.1305	619.1295	1.1	1.8	15.0000

--- End Of Report ---

Figure S2. HRMS of EGCG-G1 (**2**).

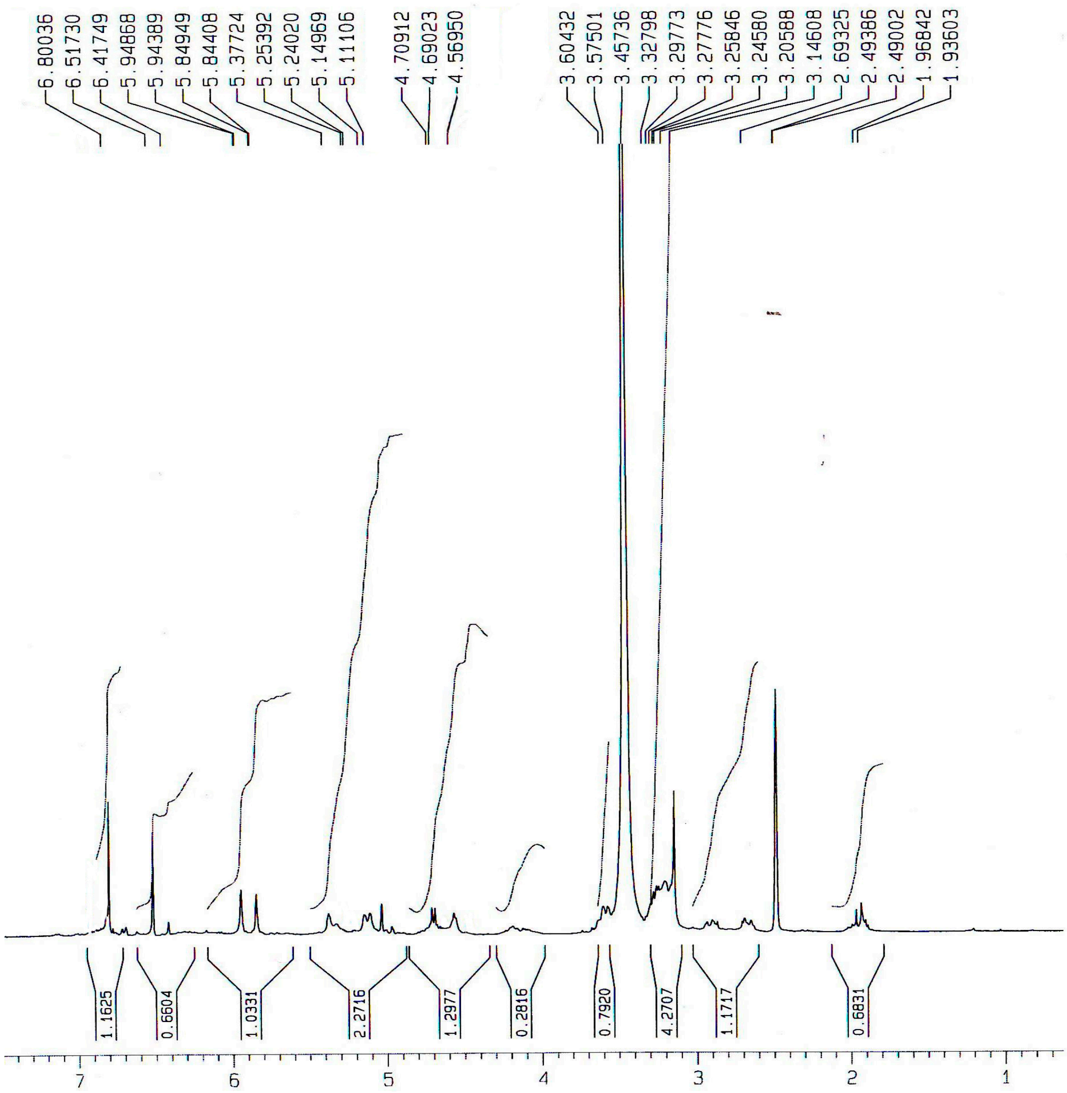

Figure S3. ^{1}H-NMR of EGCG-G1 (**2**).

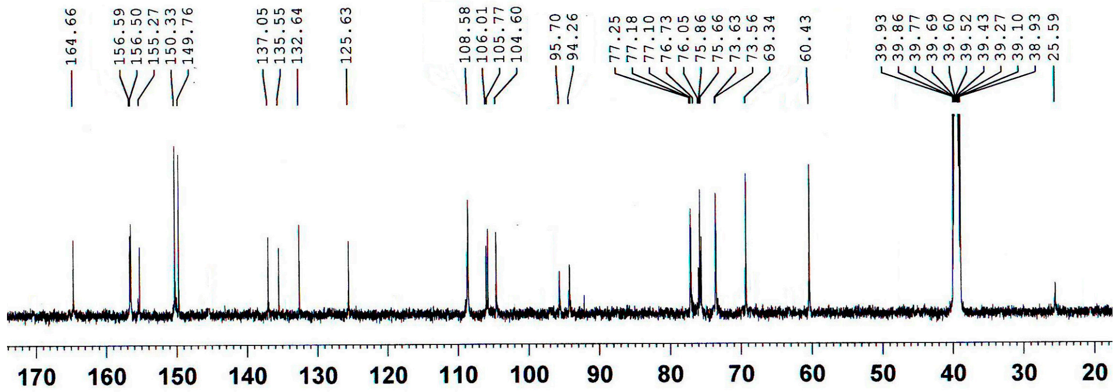

Figure S4. ^{13}C-NMR of EGCG-G1 (**2**).

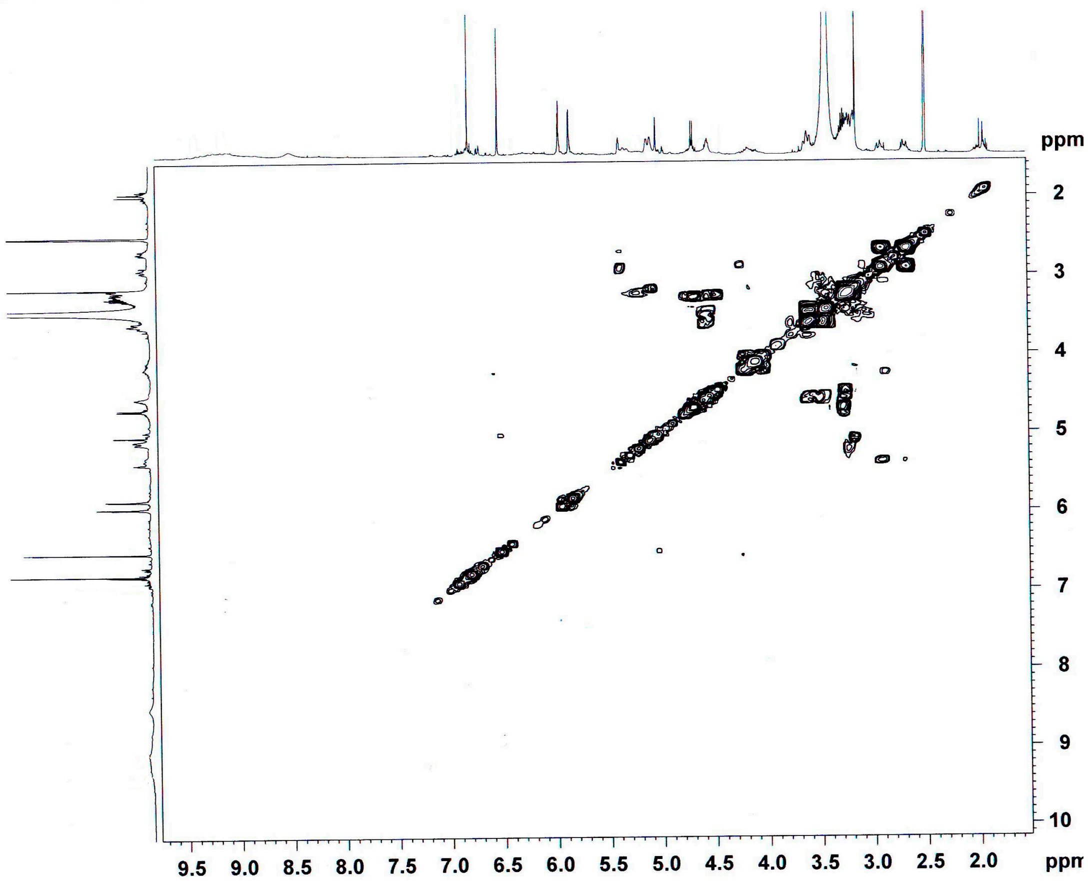

Figure S5. COSY of EGCG-G1 (**2**).

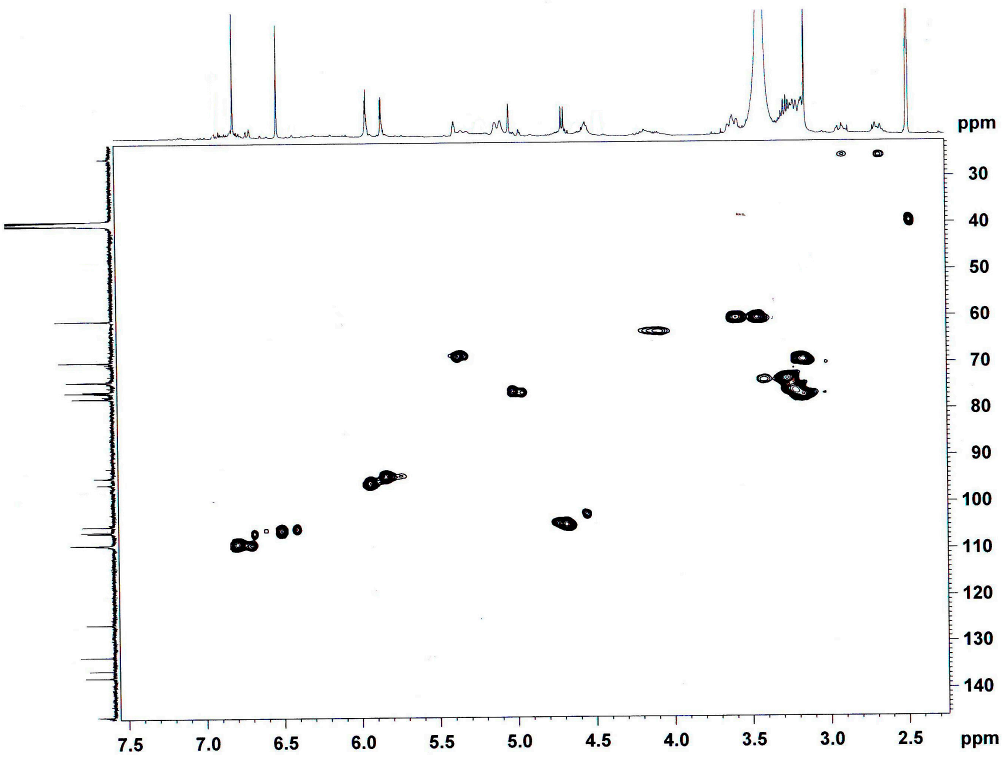

Figure S6. HSQC of EGCG-G1 (**2**).

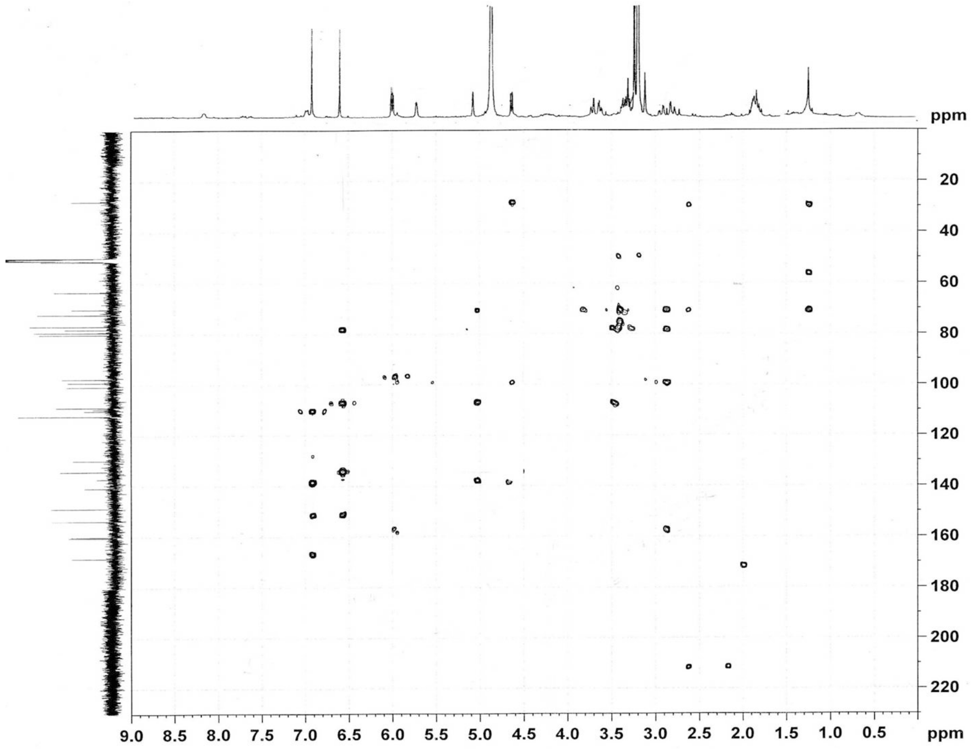

Figure S7. HMBC of EGCG-G1 (**2**).

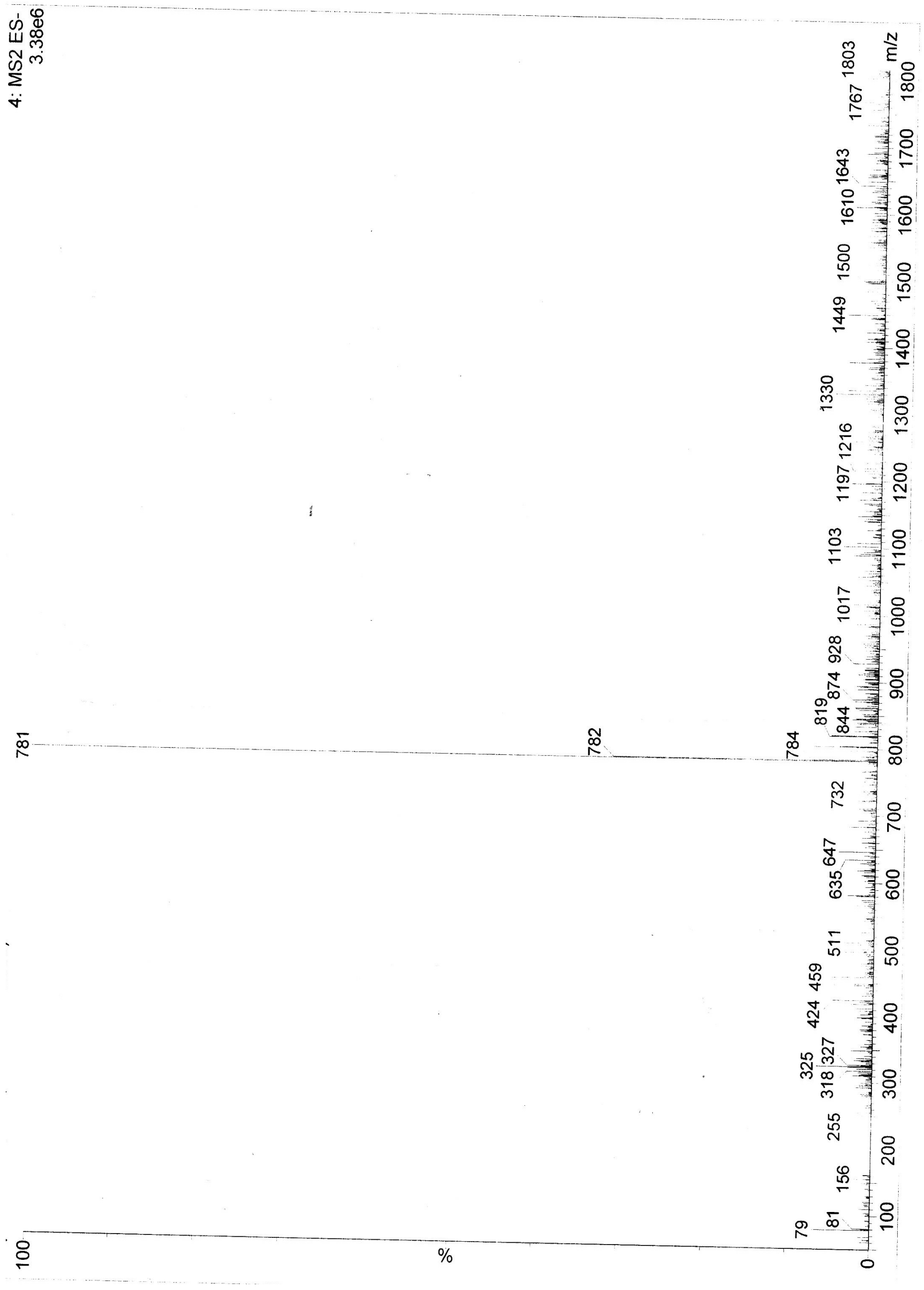

Figure S8. ESI MS of EGCG-G1 (**3**).

User Spectra

Fragmentor Voltage	Collision Energy	Ionization Mode
135	0	ESI

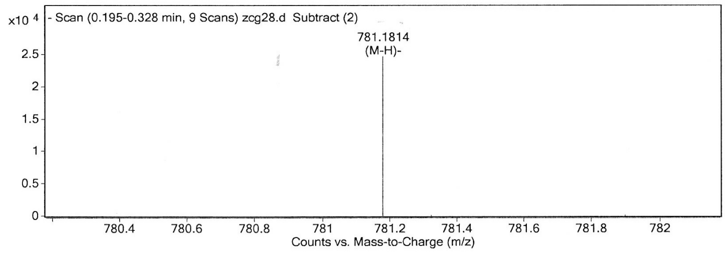

Peak List

m/z	z	Abund	Formula	Ion
781.1814	1	24798.21	C34 H38 O21	(M-H)-
782.1845	1	9102.5	C34 H38 O21	(M-H)-
817.1588	1	50449.92		
818.1617	1	18442.21		
819.1581	1	20595.71		
895.1738	1	10107.69		

Formula Calculator Element Limits

Element	Min	Max
C	3	60
H	0	120
O	0	30

Formula Calculator Results

Formula	CalculatedMass	CalculatedMz	Mz	Diff. (mDa)	Diff. (ppm)	DBE
C34 H38 O21	782.1906	781.1833	781.1814	1.9	2.5	16.0000

Figure S9. HRMS of EGCG-G2 (**3**).

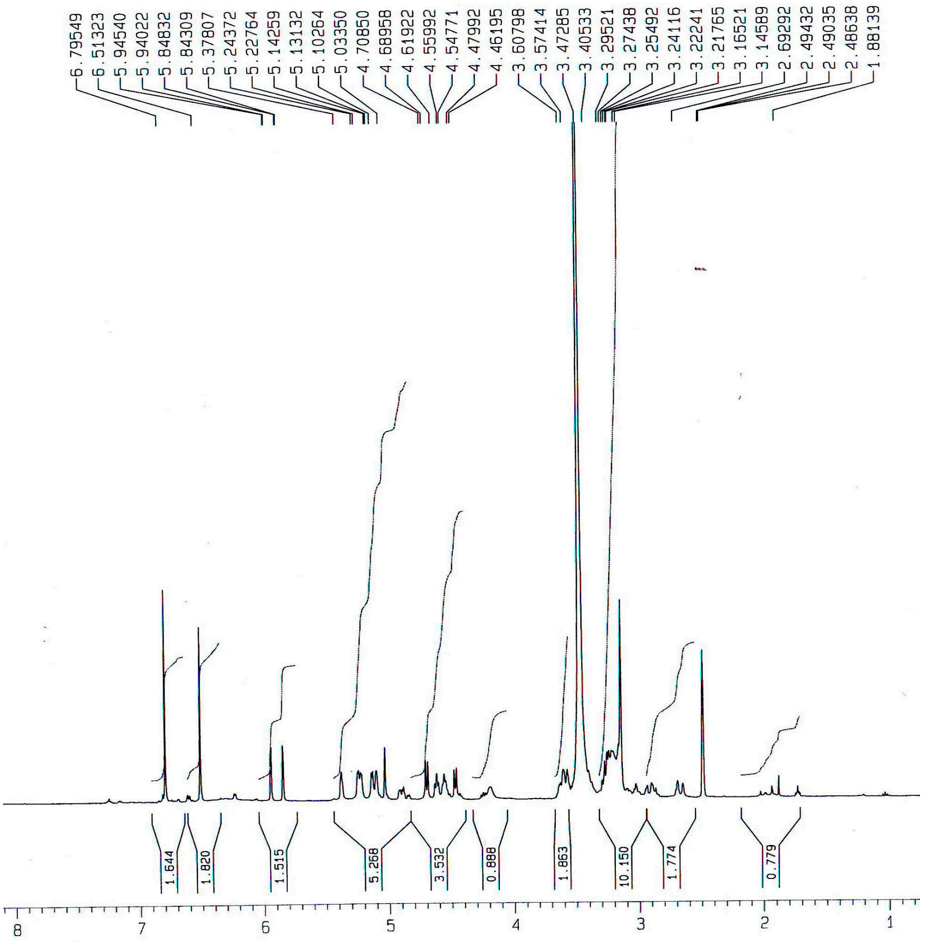

Figure S10. ^{1}H-NMR of EGCG-G2(**3**).

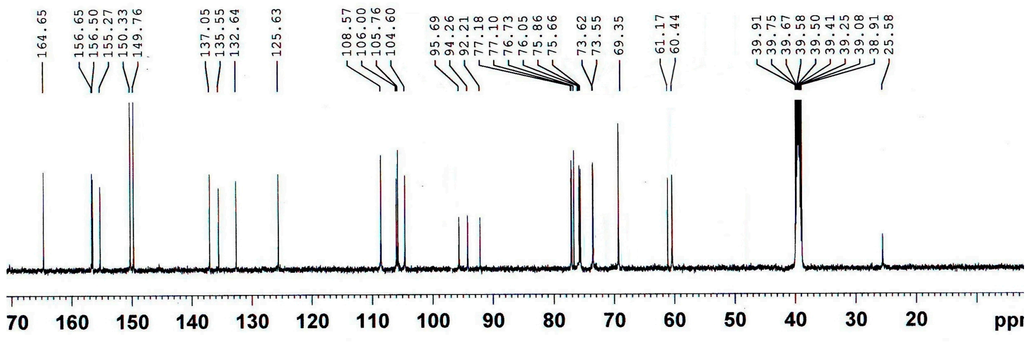

Figure S11. ^{13}C-NMR of EGCG-G2 (**3**).

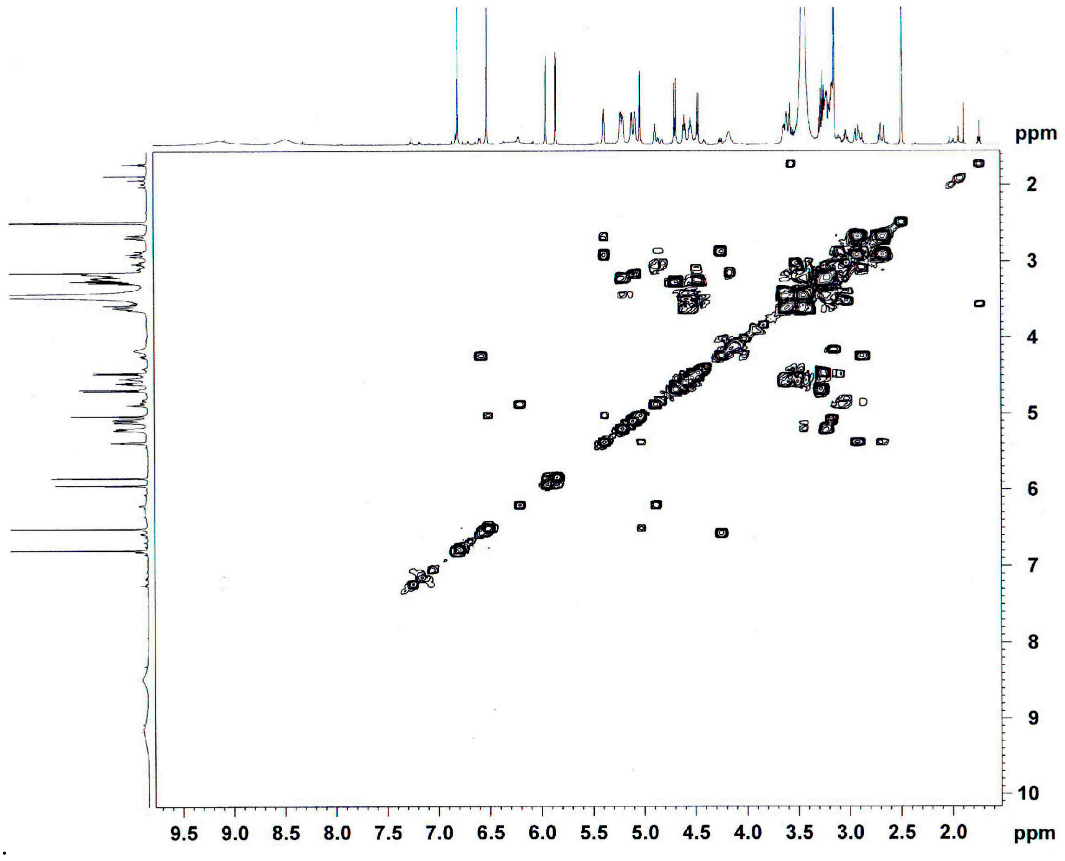

Figure S12. COSY of EGCG-G2 (**3**).

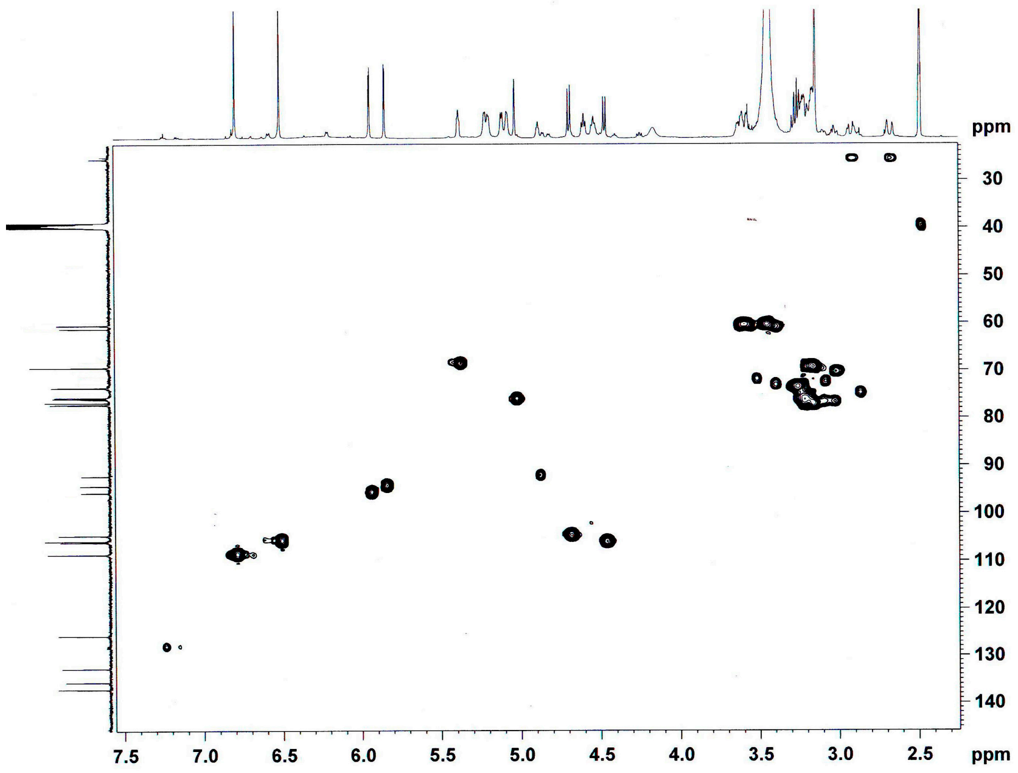

Figure S13. HSQC of EGCG-G2 (**3**).

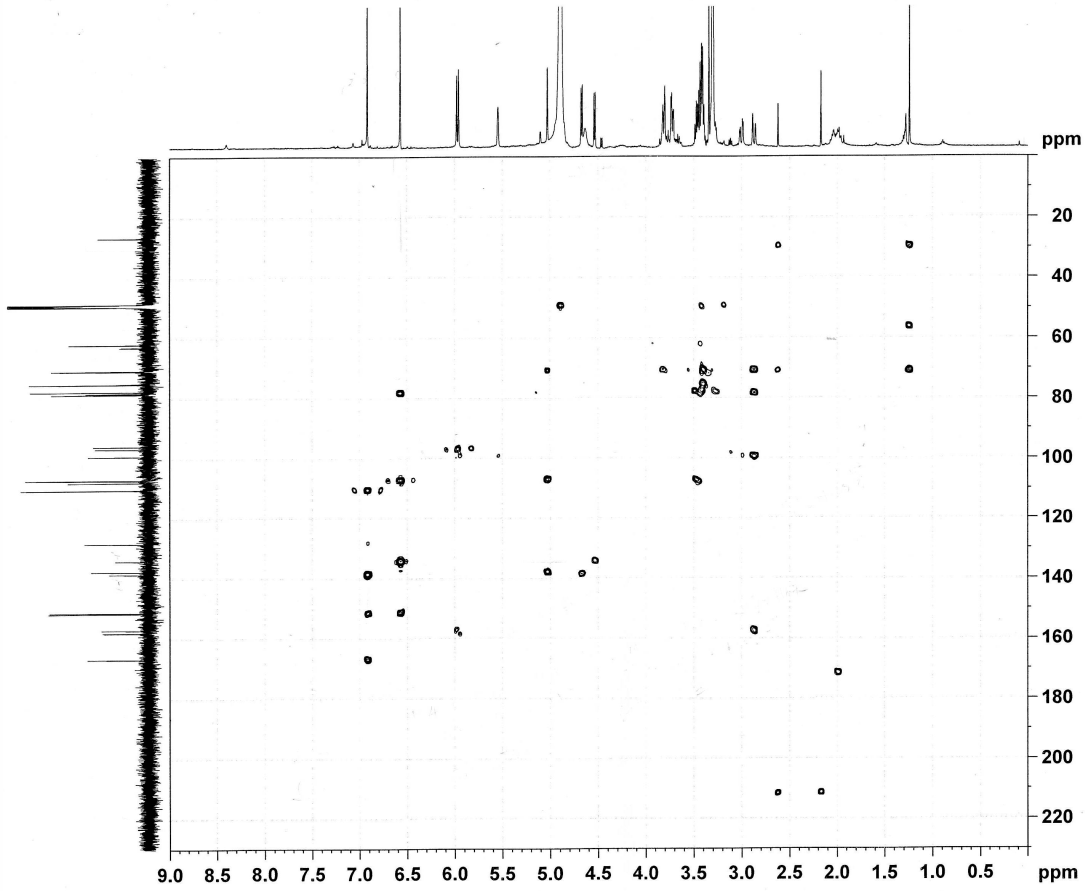

Figure S14. HMBC of EGCG-G2 (**3**).
